# Feeling Safe and Nostalgia in Healthy Aging

**DOI:** 10.3389/fpsyg.2022.843051

**Published:** 2022-04-04

**Authors:** Julie Fleury, Constantine Sedikides, Tim Wildschut, David W. Coon, Pauline Komnenich

**Affiliations:** ^1^Center for Innovation in Healthy and Resilient Aging, Edson College of Nursing and Health Innovation, Arizona State University, Phoenix, AZ, United States; ^2^Centre for Research on Self and Identity, Department of Psychology, University of Southampton, Southampton, United Kingdom

**Keywords:** feeling safe, emotion regulation, intrinsic capacity, healthy aging, healthy aging and wellbeing

## Abstract

The population of older adults worldwide is growing, with an urgent need for approaches that develop and maintain intrinsic capacity consistent with healthy aging. Theory and empirical research converge on feeling safe as central to healthy aging. However, there has been limited attention to resources that cultivate feeling safe to support healthy aging. Nostalgia, “a sentimental longing for one’s past,” is established as a source of comfort in response to social threat, existential threat, and self-threat. Drawing from extant theory and research, we build on these findings to position nostalgia as a regulatory resource that cultivates feeling safe and contributes to intrinsic capacity to support healthy aging. Using a narrative review method, we: (a) characterize feeling safe as a distinct affective dimension, (b) summarize the character of nostalgia in alignment with feeling safe, (c) propose a theoretical account of the mechanisms through which nostalgia cultivates feeling safe, (d) highlight the contribution of nostalgia to feeling safe and emotional, physiological, and behavioral regulatory capabilities in healthy aging, and (e) offer conclusions and direction for research.

## Introduction

The population of older adults worldwide is growing disproportionately. The number of people aged 60 years and older constitute 13% of the global population and is expected to double by 2050 (World Health Organization, 2020). Healthy aging, the development and maintenance of functional ability consistent with well-being in older age, is a public health priority and the primary goal of aging research (Friedman et al., 2019; Aronson, 2020). Functional ability refers to being and doing things of value, pursuing meaningful objectives, and fulfilling one’s potential with dignity (Rudnicka et al., 2020). Functional ability is determined by intrinsic capacity, the emotional, physiological, and behavioral regulatory capabilities of older adults in interaction with their environment (Cesari et al., 2018).

Theory and empirical research converge on feeling safe as essential to intrinsic capacity in healthy aging (Slavich, 2020; Porges, 2021; Thayer et al., 2021). However, there has been limited attention to resources that cultivate feeling safe to support healthy aging (Epel, 2020). Nostalgia is established as a source of comfort in response to social threat (Sedikides and Wildschut, 2019), existential threat (Sedikides and Wildschut, 2018), and self-threat (Sedikides et al., 2015). Drawing from extant theory and empirical research, we build on these findings to position nostalgia as a regulatory resource that cultivates feeling safe and contributes to intrinsic capacity to support healthy aging. Relying on a narrative review method, we: (a) characterize feeling safe as a distinct affective dimension to support healthy aging, (b) summarize the character of nostalgia in alignment with feeling safe as a distinct affective dimension, (c) propose a theoretical account of the mechanisms through which nostalgia cultivates feeling safe, (d) highlight the contribution of nostalgia to feeling safe and emotional, physiological, and behavioral regulatory capabilities to support healthy aging, and (e) offer conclusions and direction for research.

## Feeling Safe

Gilbert (2009) has proposed a tripartite model of affect comprised of negative affect, positive affect, and safeness, evolved in the context of specific environmental stimuli. Negative affect alerts to threat or danger, positive affect energizes seeking resources, and safeness reduces activation for calm and restoration. Feeling safe is characterized by warmth and affiliative connection (Gilbert, 2009, 2015). Compared with positive affect, feeling safe predicts unique variance in perceived stress, perceived social support, and depressive symptoms (McManus et al., 2019; Armstrong et al., 2021). In the autonomic nervous system, feeling safe is characterized by increased parasympathetic activity and inhibition of sympathetic response, indexed as higher vagally mediated heart rate variability (vmHRV), a measure of the parasympathetic regulation of the heart *via* the vagus nerve (McCraty, 2017; Mather and Thayer, 2018). Resting vmHRV represents the physiological and emotional regulatory capabilities of older adults in response to environmental challenges (Thayer and Lane, 2009; Thayer et al., 2012). Relevant to healthy aging, higher vmHRV is associated with improved cognitive performance (Ottaviani et al., 2019), greater capacity for emotion regulation (Mather and Thayer, 2018), and attenuated inflammation and oxidative stress (Liguori et al., 2018; Virani et al., 2020).

Feeling safe relies on learned safety cues uniquely associated with protection from threat, found in familiar patterns and coherence, continuity in sense of self and relationships, and reliable close connections (Brosschot et al., 2018). Safety cues represent sources of predictability, protection, comfort, soothing, and connection (Gee and Cohodes, 2021). From the perspective of social engagement, cues of safety include caring facial expressions, soft eye contact, warmth, and prosody of voice (Porges, 2007; Geller, 2018). Safety cues and experiences of feeling safe serve emotional and physiological regulatory functions across the lifespan (Cho et al., 2021; Gee and Cohodes, 2021). In middle-aged and older adults, memories of warmth and safeness are associated with better self-rated health and lower depressive symptoms over intervals of approximately 6 and 18 years (Chopik and Edelstein, 2019). Memories of warmth and safeness contribute to psychological and social well-being (Matos et al., 2013; Ferreira et al., 2021). In addition, memories of warmth and safeness are linked to safe affect and lower levels of anxiety and stress symptoms (Capinha et al., 2021; Steindl et al., 2021).

## Nostalgia

Nostalgia, “a sentimental longing or wistful affection for the past” (*The New Oxford Dictionary of English*, 1998, p. 1266), aligns with feeling safe as a distinct affective dimension. Nostalgia is experienced across the lifespan (Hepper et al., 2021) and across cultures and ethnicities (Hepper et al., 2012; Jenkins, 2016; Viladrich and Tagliaferro, 2016; Orr, 2017). Nostalgia is felt as positive and bittersweet, with a calming physiological and emotional response, consistent with feeling safe (Sedikides and Wildschut, 2018; Vaccaro et al., 2020). Also, nostalgia is a positively valenced, approach-oriented, and low arousal emotion, consistent with feeling safe as a distinct affective dimension (van Tilburg et al., 2018; Sedikides and Wildschut, 2020; Leunissen et al., 2021). Further, nostalgia is highly social: nostalgic memories depict the individual as having a central place in relevant events, albeit surrounded by close others (Sedikides and Wildschut, 2019). The social nature of nostalgia aligns with the affiliative emotion regulation system, that is, detecting and responding to safety cues by increasing parasympathetic response to facilitate calm and quiescence (Abeyta et al., 2020). Moreover, multidimensional scaling analyses emphasize the similarity of nostalgia to prosocial emotions such as self-compassion (van Tilburg et al., 2018), which is associated with higher vmHRV (Di Bello et al., 2020).

Nostalgia entails a sense of coherence, fostering predictability and close connections (Synnes, 2015). Sense of coherence is a key determinant of well-being and reduced distress in older adults (del-Pino-Casado et al., 2019). Furthermore, nostalgia promotes self-continuity, a connection between past and present selves (Sedikides et al., 2016), which is essential for positive function in later life (Löckenhoff and Rutt, 2017). Among older adults with mild to moderate levels of dementia, nostalgia improves the recall and recognition of self-referent information (Ismail et al., 2018). Moreover, nostalgia strengthens social connection and re-experiencing relational bonds in close relationships (Juhl et al., 2020), and offsets loneliness by increasing perceived social support (Frankenbach et al., 2021; Zhou et al., 2021; Wildschut and Sedikides, 2022).

## Mechanisms of Action

Nostalgia engages safety cues, thereby cultivating feeling safe and contributing to regulatory capabilities to support healthy aging. Prefrontal-subcortical inhibitory pathways in the brain enact the regulatory response to threat and safety and are linked to the heart *via* the vagus nerve (Thayer and Lane, 2009; Thayer et al., 2012). The neural circuits in the regulatory response to threat and safety include the amygdala, which detects emotionally salient stimuli in the environment; the hippocampus, which is involved in learning and memory; and the medial prefrontal cortex (mPFC), which regulates reactivity of the amygdala (Thayer et al., 2012; Dolcos et al., 2017; Eichenbaum, 2017; Gee and Cohodes, 2021). Brosschot et al. (2018) propose that the default response in humans is threat or defense, with prolonged sympathetic nervous system activity and associated chronic illness (Thayer and Lane, 2009; Thayer et al., 2012). The threat response is active absent recognized safety; safety cues inhibit the default threat response (Brosschot et al., 2018). Engaging safety cues inhibits the amygdala through the input of the ventromedial prefrontal cortex (vmPFC) and hippocampus, with increased parasympathetic activity and higher vmHRV, consistent with feeling safe (Thayer et al., 2009; Brosschot et al., 2018). Higher vmHRV in feeling safe contributes to emotional, physiological, and behavioral regulatory capabilities (Smith et al., 2017; Mather and Thayer, 2018).

## Feeling Safe and Nostalgia in Healthy Aging

Healthy aging reflects the emotional, physiological, and behavioral regulatory capabilities of older adults in interaction with their environment (Cesari et al., 2018). For most adults, aging is associated with changes in the brain and the heart which limit regulatory capabilities in response to environmental challenges (Thayer and Lane, 2009; Thayer et al., 2012). Concomitant changes in the brain and the heart with aging appear to be associated with a decrease in PFC inhibition of the default threat response, autonomic imbalance, and associated chronic illness (Thayer and Lane, 2009; Thayer et al., 2012). Thus, approaches which support the brain-heart regulatory response to threat and safety in older adulthood are essential (Thayer et al., 2021). While research addressing nostalgia in older adults is limited, nostalgia may be especially relevant in older age given its potential to cultivate feeling safe and contribute to regulatory capabilities.

## Emotional Regulatory Capabilities

Nostalgia contributes to emotional regulatory capabilities that may support healthy aging. Among older adults, nostalgia provides a safe haven in the face of adversity (Madoglou et al., 2017). Nostalgia augments comfort and security (Walls, 2021), and maintains psychological well-being when confronted with limited time horizons (Hepper et al., 2021). During COVID-19 pandemic restrictions, nostalgic memories provided solace to older adults in the context of uncertainty and change (Huntley and Bratt, 2022). In those with dementia, engaging in nostalgia increased self-esteem, meaning in life, and positive affect (Ismail et al., 2018). Similarly, an intervention evaluating the psychological benefits of nostalgic conversations in people living with mild-to-moderate dementia and their partners reported improved self-esteem, personal growth, meaning in life, and social connectedness, with the strongest evidence for improvement shown in personal growth for the person with dementia (Dodd et al., 2021). Among older adults near the end of life, nostalgia supports affective meaning and fosters a sense of being loved and protected (Synnes, 2015). Further, nostalgic memories engender restoration, a soothing sense of being at home (Missel et al., 2022).

## Physiological Regulatory Capabilities

Nostalgia contributes to physiological regulatory capabilities to support healthy aging. Wu and Chang (2017) evaluated the effects of recalling a nostalgic memory relative to a recent general memory on emotional and autonomic response in older adults. Compared with the control condition, older adults recalling a nostalgic memory showed a pattern of autonomic inhibition with increased parasympathetic activity. Fu et al. (2018) evaluated the effects of discussing nostalgic smells, relative to discussing general themes on emotional and physiological outcomes among older adults in long-term care. Compared with the control condition, older adults discussing nostalgic smells showed decreased symptoms of anxiety and depression and increased HRV. Also, Suenaga et al. (2018) evaluated the effect of nostalgic, relative to general images on vmHRV in older adults. Nostalgic images increased feelings of relaxation and increased vmHRV. Rasmussen et al. (2021) used nostalgic films to elicit involuntary episodic memories and emotional response in older adults with Alzheimer’s disease and healthy controls. Older adults with Alzheimer’s disease experienced relatively more episodic memories and greater emotional response to nostalgic films. These findings align with research evaluating the psychological and physiological response associated with odor-evoked nostalgia in younger adults, in which odor-evoked nostalgic memories elicited greater positive affect, reduced symptoms of anxiety and depression, decreased heart rate, inhibited systemic inflammation, when compared with control odors (Matsunaga et al., 2011, 2013).

## Behavioral Regulatory Capabilities

Nostalgia contributes to behavioral regulatory capabilities that support healthy aging. Nostalgia strengthens motivation to promote or maintain social and physical functioning essential for well-being in later adulthood. Nostalgia provides a context for social interaction, which may be particularly important for socially isolated older adults. In older adults with mild-to-moderate dementia in long term care, nostalgia fostered social connections and inter-personal communication (Irmanti and Wardono, 2021). These findings align with research in younger adults, in which nostalgia increased commitment to approach-oriented social goals such as getting closer to friends, repairing relationship conflicts, and meeting new people (Abeyta et al., 2015). Nostalgia fosters motivation for physical activity in older adults by linking the past to the present. For example, in older adults with cognitive impairment, nostalgic memories supported the intention to engage in a program of social dance (Thøgersen-Ntoumani et al., 2018). Nostalgic memories of past gardens and gardening with parents and grandparents were evoked through the touch and smell of plants, soil, and herbs, and enacted through gardening as a means of tradition, remembrance, and connection (McFarland et al., 2018; Scott et al., 2020).

## Discussion

The population of older adults worldwide is growing, with an urgent need to develop and maintain intrinsic capacity to support healthy aging (Friedman et al., 2019). Theory and empirical research converge on feeling safe as essential to intrinsic capacity in healthy aging (Slavich, 2020; Porges, 2021; Thayer et al., 2021). However, there has been limited attention to resources that cultivate feeling safe (Epel, 2020). Drawing from extant theory and empirical research, we position nostalgia as a regulatory resource that cultivates feeling safe and contributes to intrinsic capacity to support healthy aging.

As an approach to intervention supporting healthy aging, nostalgia may have promise across older adult populations. Nostalgia aligns with feeling safe as a distinct affective dimension, providing a novel perspective on cultivating sources of safety in older adulthood rather than managing sources of threat. Cognitive-behavioral, reappraisal, or coping interventions are focused on managing the “why” of threats to feeling safe, mobilizing the default response, with increased sympathetic nervous system activity and risk of chronic illness (Thayer and Lane, 2009; Thayer et al., 2012). In contrast, nostalgia is focused on the experiential “what” of feeling safe, calming physiological and emotional response. A theoretical account of the mechanisms through which nostalgia cultivates feeling safe reinforces the potential for nostalgia as a regulatory practice. Consistent with the proposed mechanisms of action, engaging safety cues in nostalgia may enhance functional connectivity between the vmPFC and the amygdala and strengthen inhibition of the default threat response, with cumulative effects of preserving intrinsic capacity and long-term well-being.

Nostalgia contributes to emotional, physiological, and behavioral regulatory capabilities; this regulatory quality may be vital in distinguishing nostalgia from traditional autobiographical approaches in gerontology. Nostalgia may be especially relevant as an approach to intervention in older adulthood, given its potential to support the brain-heart regulatory response in the context of threat and safety.

Several issues deserve empirical consideration. For example, research is needed to determine the amount and duration of nostalgia necessary to cultivate feeling safe and contribute to regulatory capabilities. Longitudinal research is needed to determine if changes in emotional, physiological, or behavioral regulatory capabilities in response to nostalgia are sustained. Comparative effectiveness trials can establish which approaches to nostalgia induction are most effective, for whom, and under what conditions. Given the emphasis on healthy aging, future studies might test whether changes in neural and autonomic function with age introduce variability in response to nostalgia.

Nostalgia is a precious resource for older adults, as it can be accessed at any time, even when social opportunities are limited (Hepper et al., 2021). Older adults face physical and emotional challenges in pursuit of healthy aging. However, many also possess rich nostalgic memories to cultivate feeling safe in support of healthy aging. In nostalgia, older adults navigate the future by reflecting on the past. By doing so, they find safety in sources such as familiar patterns and coherence, continuity in the sense of self or relationships, and affectionate close bonds.

## Author Contributions

JF, DWC, and PK: conceptualization. JF, CS, and TW: methodology, analysis, and writing initial drafts. JF, CS, TW, DWC, and PK: review and editing. All authors contributed to the article and approved the submitted version.

## Conflict of Interest

The authors declare that the research was conducted in the absence of any commercial or financial relationships that could be construed as a potential conflict of interest.

## Publisher’s Note

All claims expressed in this article are solely those of the authors and do not necessarily represent those of their affiliated organizations, or those of the publisher, the editors and the reviewers. Any product that may be evaluated in this article, or claim that may be made by its manufacturer, is not guaranteed or endorsed by the publisher.
